# Lobular breast cancer - the most common special subtype or a most special common subtype?

**DOI:** 10.1186/s13058-015-0606-z

**Published:** 2015-07-28

**Authors:** Ulrich Lehmann

**Affiliations:** Institute of Pathology, Medizinische Hochschule Hannover, Carl-Neuberg-Str. 1, D-30625 Hannover, Germany

## Abstract

Lobular breast cancer is not only the second most common breast cancer subtype, known for decades, but also a tumour entity that still poses many unresolved questions. These include questions about the targets and cooperation partners of E-cadherin, the best model systems for translational research, and the best tools for detection, surveillance and therapy. Leading experts review the molecular and cellular bases, the model systems, the histopathology and profiling approaches, risk factors, imaging tools and therapeutic options for lobular breast cancer.

## ᅟ

Numerous commentaries or reviews about lobular breast cancer begin with the phrase 'the most frequent special subtype of human breast cancer' or some variation of it. Often the statement follows that lobular breast cancer typically has (whichever percentage of cases is hidden behind the word 'typically') a better prognosis. However, gynaecological oncologists from time to time see patients with a reasonably small, nearly indolent looking breast tumour classified by the pathologist as 'lobular' which, at some point, develops into a highly aggressive, principally unmanageable metastatic disease. As well as this, some might say 'anecdotal', evidence contradicting the usual phrase about the 'typically better prognosis', comprehensive studies on really large cohorts also do not support this view [1]. The long-term prognosis might be even worse compared with invasive ductal carcinoma [2]. But what are the underlying molecular mechanisms?

A totally different peculiar feature of lobular breast cancer is the near universal loss of the cell adhesion protein E-cadherin due to deletion, mutation or hypermethylation of the *CDH1* gene promoter. More than a few people in the field argue that loss of E-cadherin staining together with the characteristic growth pattern are defining characteristics of lobular breast cancer, a unique association between histology and genetics in breast cancer. However, this view is contested by the, admittedly small, group of E-cadherin-negative *bona fide* invasive-ductal breast carcinomas (see McCart Reed and colleagues in this series [3]).

A puzzling observation concerning the importance of E-cadherin in lobular breast cancer, however, is the absence of tumour formation in conditional *CDH1* knock-out models (see Christgen and Derksen in this series [4]), leaving plenty of room for speculation about the 'real' oncogenic hit in lobular breast cancer, the alterations 'upstream' of E-cadherin and the contribution of E-cadherin loss to the development and progression of lobular breast cancer.

The peculiar growth pattern (due to loss of proper cell-cell contacts?) directly translates into challenges for the clinical detection of lobular breast cancer by physical examination or imaging because in many patients the tumour mass is so diffuse that it might evade timely and reliable detection (see Johnson and colleagues in this series [5]).

The specific growth pattern mentioned above links the molecular and cellular foundations of the biology of lobular breast cancer cells not only to challenges in detection but also to controversies and discussions about the most efficient treatment strategies. These discussions and decisions about surgical intervention, radiation protocols and systemic therapy are also influenced and informed by our growing knowledge about the relationship between early changes in morphology ('lobular neoplasia', 'lobular intraepithelial neoplasia', 'lobular carcinoma *in situ*' and so on) and overt invasive malignancy and the underlying driver mutations (see Logan and colleagues in this series [6]).

Despite the very strong, albeit not 100 % perfect, correlation between loss of E-cadherin protein expression and the lobular subtype, the relationship between *CDH1* germline mutations and the risk of lobular breast cancer development is much more complex (see Dossus and Benusiglio in this series [7]).

A further unresolved topic regarding invasive lobular breast cancer is the contradiction between the very high proportion of estrogen receptor (ER)-positive lobular breast cancer specimens (more than 90 % depending on the study) and the comparably low efficiency of anti-estrogen therapy in this patient cohort compared with invasive ductal breast cancer with a much lower proportion of ER-positive specimens (for example, [8]). This is not properly understood on the molecular level, but is of real importance in clinics. Comprehensive sequence analyses of the estrogen receptor gene and genes encoding ER pathway components in appropriately sized patient cohorts might contribute to the identification of clinically relevant mutations conferring endocrine resistance while still providing proper immunohistochemical staining scored as 'ER positive' ([9] and references therein).

These observations and clinical experiences hint at the fact that 'lobular breast cancer' is far less understood than many readers of *Breast Cancer Research* might assume. Therefore, a timely update on the molecular and cellular bases of this somewhat elusive disease, proper model systems to study it, genetic and non-genetic risk factors causing it, methods to detect it, and protocols to treat it seems to be warranted.

## Note

This article is part of a series on Lobular breast cancer, edited by Ulrich Lehmann. Other articles in this series can be found at http://breast-cancerresearch.com/series/LBC

## Abbreviation

ER: estrogen receptor
